# An Incompatibility between a Mitochondrial tRNA and Its Nuclear-Encoded tRNA Synthetase Compromises Development and Fitness in *Drosophila*

**DOI:** 10.1371/journal.pgen.1003238

**Published:** 2013-01-31

**Authors:** Colin D. Meiklejohn, Marissa A. Holmbeck, Mohammad A. Siddiq, Dawn N. Abt, David M. Rand, Kristi L. Montooth

**Affiliations:** 1 Department of Ecology and Evolutionary Biology, Brown University, Providence, Rhode Island, United States of America; 2 Department of Molecular Biology, Cell Biology, and Biochemistry, Brown University, Providence, Rhode Island, United States of America; 3 Department of Biology, Indiana University, Bloomington, Indiana, United States of America; University of Wisconsin–Madison, United States of America

## Abstract

Mitochondrial transcription, translation, and respiration require interactions between genes encoded in two distinct genomes, generating the potential for mutations in nuclear and mitochondrial genomes to interact epistatically and cause incompatibilities that decrease fitness. Mitochondrial-nuclear epistasis for fitness has been documented within and between populations and species of diverse taxa, but rarely has the genetic or mechanistic basis of these mitochondrial–nuclear interactions been elucidated, limiting our understanding of which genes harbor variants causing mitochondrial–nuclear disruption and of the pathways and processes that are impacted by mitochondrial–nuclear coevolution. Here we identify an amino acid polymorphism in the *Drosophila melanogaster* nuclear-encoded mitochondrial tyrosyl–tRNA synthetase that interacts epistatically with a polymorphism in the *D. simulans* mitochondrial-encoded *tRNA^Tyr^* to significantly delay development, compromise bristle formation, and decrease fecundity. The incompatible genotype specifically decreases the activities of oxidative phosphorylation complexes I, III, and IV that contain mitochondrial-encoded subunits. Combined with the identity of the interacting alleles, this pattern indicates that mitochondrial protein translation is affected by this interaction. Our findings suggest that interactions between mitochondrial tRNAs and their nuclear-encoded tRNA synthetases may be targets of compensatory molecular evolution. Human mitochondrial diseases are often genetically complex and variable in penetrance, and the mitochondrial–nuclear interaction we document provides a plausible mechanism to explain this complexity.

## Author Summary

The ancient symbiosis between two prokaryotes that gave rise to the eukaryotic cell has required genomic cooperation for at least a billion years. Eukaryotic cells respire through the coordinated expression of their nuclear and mitochondrial genomes, both of which encode the proteins and RNAs required for mitochondrial transcription, translation, and aerobic respiration. Genetic interactions between these genomes are hypothesized to influence the effects of mitochondrial mutations on disease and drive mitochondrial–nuclear coevolution. Here we characterize the molecular cause and the cellular and organismal consequences of a mitochondrial–nuclear interaction in *Drosophila* between naturally occurring mutations in a mitochondrial tRNA and a nuclear-encoded tRNA synthetase. These mutations have little effect on their own; but, when combined, they severely compromise development and reproduction. tRNA synthetases attach the appropriate amino acid onto their cognate tRNA, and this reaction is required for efficient and accurate protein synthesis. We show that disruption of this interaction compromises mitochondrial function, providing hypotheses for the variable penetrance of diseases associated with mitochondrial tRNAs and for which pathways and processes are likely to be affected by mitochondrial–nuclear interactions.

## Introduction

Aerobic eukaryotes fuel development, physiological performance and reproduction via the coordinated expression of their mitochondrial and nuclear genomes. Mitochondrial replication, transcription, translation and respiration depend upon interactions between RNAs and proteins encoded in both genomes, and epistatic interactions between mitochondrial and nuclear polymorphisms are known to contribute to phenotypic and fitness variation within species [1]–[6]. As a consequence of mitochondrial-nuclear fitness interactions, coadapted or compensatory substitutions are predicted to accumulate between coevolving mitochondrial and nuclear genomes as lineages diverge [6]–[10]. Consistent with this prediction, the fitness consequences of combining foreign nuclear and mitochondrial genomes are greater among more divergent populations of the same species [1]–[3], [11]. Insight into the molecular mechanisms underlying mitochondrial-nuclear interactions, however, have come largely from investigation of incompatibilities between mitochondrial and nuclear genomes from very divergent populations or between closely related species [6], [11]–[16], as incompatibilities with large fitness effects are unlikely to be segregating within a population.

The majority of the animal mtDNA encodes protein subunits of the oxidative phosphorylation (OXPHOS) complexes, and these complexes are comprised of proteins encoded in both the mitochondrial and nuclear genomes, with the exception of Complex II, for which all subunits are encoded by nuclear loci. Not surprisingly, mitochondrial-nuclear incompatibilities have been shown to compromise this core function of the mitochondrial genome [11], [12], [17]. Interactions between the nuclear-encoded cytochrome *c* and the mitochondrial-encoded subunits of cytochrome *c* oxidase (Complex IV) reduce Complex IV activity in hybrids between divergent populations of the marine copepod *Tigriopus* [18], [19], and there is evidence that similar interactions have driven the molecular coevolution of mitochondrial- and nuclear-encoded subunits of Complex IV in primate lineages [9], [20], [21]. Osada and Akashi [9] provide compelling evidence that the elevated rate of adaptive evolution in the nuclear-encoded subunits of Complex IV is due to the fixation of compensatory substitutions driven by the fixation of deleterious mtDNA mutations, likely as a result of the increased mutation rates and lack of recombination in animal mtDNAs [22]–[24].

Mitochondrial-nuclear incompatibilities can also disrupt transcription and translation of the mitochondrial genome. Interpopulation hybrids of the marine copepod *Tigriopus* have decreased activity of all OXPHOS complexes except Complex II, which is the only complex lacking mitochondrial-encoded subunits [25]. Ellison & Burton [25], [26] hypothesize that this incompatibility results from a disrupted interaction between the nuclear-encoded mitochondrial RNA polymerase and the mtDNA control region. Mitochondrial-nuclear incompatibilities between *Saccharomyces* species also disrupt the regulation of mitochondrial gene expression, but at specific loci. Sterile F2 hybrids between *Saccharomyces cerevisiae* and either *S. paradoxis* or *S. bayanus* fail to properly splice the mitochondrial-encoded *COX1* via an interaction with the nuclear gene *Mrs1* [15]. An additional incompatibility between *S. cerevisiae* and *S. bayanus* affects translation of the *OLI1* mitochondrial mRNA due to interactions between the 5′-UTR of this gene and the nuclear gene *Aep2* [14]. Given that a third of the mitochondrial genome and a large proportion of mitochondrially-targeted nuclear gene products function in the replication, transcription and translation of the mitochondrial genome, these conserved functions are a large mutational target for generating mitochondrial-nuclear epistasis for fitness [6].

These mitochondrial-nuclear incompatibilities provide the first indications of the cellular processes disrupted by these interactions in natural populations, the genes that will experience coevolution between genomes, and the pathways that will accumulate incompatibilities as lineages diverge. However, a largely overlooked class of mitochondrial-nuclear interactions involves mitochondrial-encoded tRNAs and nuclear-encoded mitochondrial aminoacyl-tRNA synthetases (mt-aaRSs) [6]. mt-aaRSs are translated in the cytoplasm and imported to the mitochondria where they recognize and activate their cognate mt-tRNAs with the appropriate amino acid during mitochondrial protein synthesis. Mutations in mitochondrial tRNA genes can impair tRNA stability, processing and aminoacylation by mt-aaRSs [27], [28]. There are hundreds of mitochondrial tRNA mutations associated with human disease [29], as well as at least 12 known mt-aaRS disease mutations [30]. Many mt-tRNA disease mutations have complex and variable penetrance even when homoplasmic [27], [28], [31]. While there are many possible explanations for variable penetrance of mt-tRNA mutations, one hypothesis is that interactions between mitochondrial mutations and nuclear variants modify their effect on disease phenotypes [31]–[33]. Thus, tractable models of mitochondrial-nuclear interactions should advance our understanding of the genetic and physiological architecture of mitochondrial disease.

As an evolutionary screen for mitochondrial-nuclear incompatibilities, we previously substituted divergent mtDNAs from closely related *Drosophila* species into two *D. melanogaster* wild-type nuclear backgrounds (*OreR* and *AutW132*) [16]. While these *Drosophila* lineages have not accumulated fixed mitochondrial-nuclear incompatibilities, polymorphisms within these lineages generate significant mitochondrial-nuclear epistasis for fitness. One particular mitochondrial-nuclear genotype – the *D. simulans simw^501^* mtDNA in combination with the *D. melanogaster OreR* nuclear genome – has strong deleterious effects on larval-to-adult competitive fitness [16] and provides an opportunity to dissect the genetic architecture of a mitochondrial-nuclear incompatibility. Here we characterize multiple deleterious effects of this incompatibility on development and reproduction, and identify the causal interaction between single nucleotide polymorphisms (SNPs) in the mitochondrial tRNA^Tyr^ and its nuclear-encoded mt-TyrRS.

## Results

### Mitochondrial-Nuclear Epistasis Affects Development, Reproduction, and Sensory Structures

**Figure 1:**
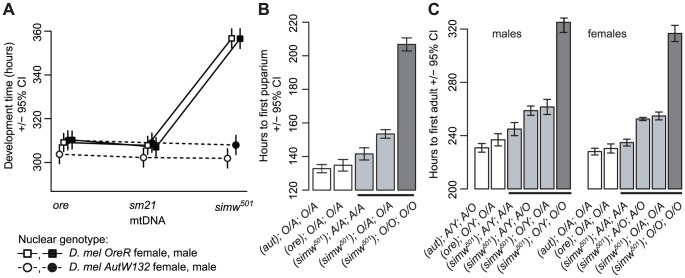
Effects of a mitochondrial–nuclear interaction on development. (A) Mitochondrial-nuclear interactions between the *D. simulans simw^501^* mtDNA and the *D. melanogaster OreR* nuclear genome significantly extend egg-to-adult development time in both sexes (mtDNA×nuclear interaction: *P_ANOVA_*≤0.0001, Table S1). The *D. simulans sm21* mtDNA is closely related to *simw^501^*, but has no effect on development time relative to the *D. melanogaster ore* mtDNA. (B and C) Both larval development and metamorphosis are delayed in *(simw^501^);OreR*. Crosses between mitochondrial-nuclear genotypes indicated that the *OreR* nuclear effect on development is similar in males and females, autosomal and largely recessive (*h* = 0.18, 0.19 and 0.23, for pupation time, and male and female eclosion times, respectively, where *h* = 0 is complete dominance of *AutW132* and *h* = 0.5 is additivity). Listed below the graphs are the (*mtDNA*);*sex chromosome*;*autosome* genotypes (*O* = *OreR*, *A* = *AutW132*). *O/A* and *A/O* heterozygotes indicate the offspring of reciprocal crosses and differ in the parent-of-origin of the autosomes (*maternal*/*paternal*). The difference in time from egg to pupation between (*simw^501^*);*AutW132* and (*simw^501^*);*OreR* is approximately 65 hours. The difference in time from egg to adult emergence between (*simw^501^*);*AutW132* and (*simw^501^*);*OreR* is 80 and 82 hours in males and females, respectively. The 65 hour delay in larval development and the additional 15–17 hour delay during metamorphosis between (*simw^501^*);*AutW132* and (*simw^501^*);*OreR* are both statistically significant (*P*_t-test_<0.001).

**Figure 2:**
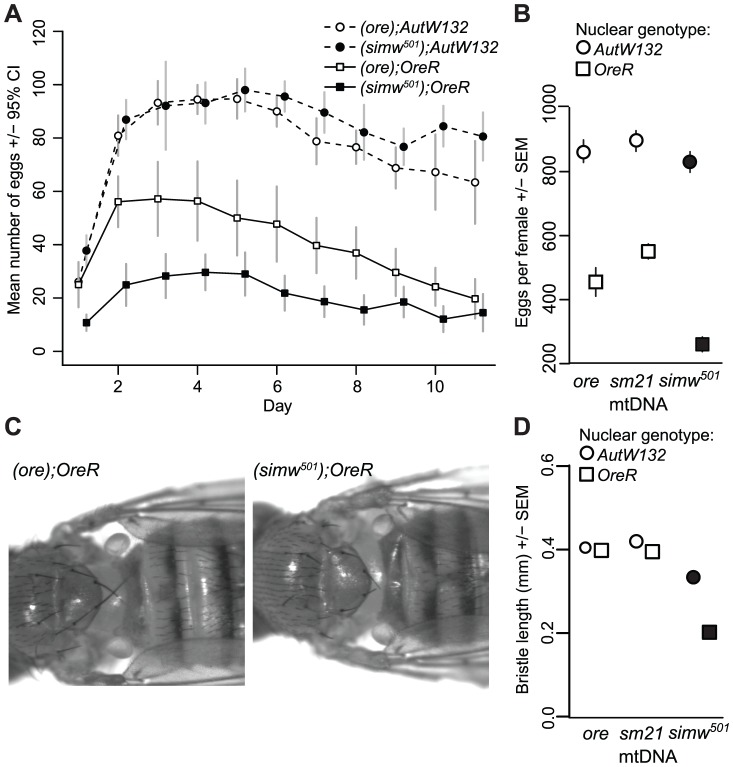
Effects of a mitochondrial–nuclear interaction on adult fecundity and sensory structures. (A) The *simw^501^* mtDNA decreases the total number of eggs females laid by 50% only in the *OreR* nuclear background (mtDNA×nuclear interaction: *F* = 9.772, *P* = 0.004, *N* = 6–11 females per genotype). There is a main effect of the nuclear genome on fecundity, presumably because *OreR* and *AutW132* are from different populations and differ at thousands of loci across their genomes. (B) A second experiment reveals the same significant mitochondrial-nuclear interaction (*P_ANOVA_* = 0.001, Table S1) and also shows that the closely related *D. simulans* mtDNA *sm21* does not decrease fecundity in either nuclear background. (C) The *simw^501^* mtDNA shortens adult mechanosensory bristles by 50% in the *OreR* nuclear background. (D) Measurement of the posterior scutellar bristles reveals a significant mitochondrial-nuclear interaction effect on bristle length (*P_ANOVA_*≤0.001, Table S1). There was no sex-by-genotype interaction, and sexes are pooled in this plot. Some error bars are smaller than the symbols.

The six mitochondrial-nuclear hybrid strains used in this study combine two inbred, wildtype *D. melanogaster* nuclear genomes, *OreR* and *AutW132*, with either the *D. melanogaster OreR* mtDNA (*ore*) or one of two *D. simulans* mtDNAs (*sm21* or *simw^501^*) [16]. We previously showed that larval-to-adult competitive fitness is decreased when the *D. simulans simw^501^* mtDNA is placed in the *OreR*, but not in the *AutW132* nuclear background [16]. The incompatible mitochondrial-nuclear genotype – hereafter denoted as (*simw^501^*);*OreR* – delays egg-to-adult development time by approximately two days via an extension of both larval development time and pupal metamorphosis (Figure 1), decreases female fecundity by 50% (Figure 2A and 2B), and shortens the adult thoracic mechanosensory bristles (Figure 2C and 2D). However, the *simw^501^* mtDNA has little to no effect on development time, fecundity or bristle length in the *AutW132* nuclear genetic background (Figure 1A and Figure 2), indicative of a significant epistatic interaction between mitochondrial and nuclear genomes (mtDNA x nuclear genome: development time, *F_2,164_* = 169.5, *P*<0.0001; fecundity, *F_2,60_* = 7.33, *P* = 0.0014; bristle length, *F_2,126_* = 250.3, *P*<0.0001; Table S1). The closely related *D. simulans sm21* mtDNA has no detectable deleterious effect on development time, female fecundity or bristle length in either nuclear background, indicating that it is largely compatible with the *D. melanogaster* nuclear genome (Figure 1A and Figure 2B and 2D). This shows that the >600 fixed nucleotide differences between *D. melanogaster* and *D. simulans* mtDNAs [34], [35] do not cause this interaction and implicates mutations unique to the *simw^501^* mtDNA.

### Genetic Architecture of the Mitochondrial–Nuclear Interaction

**Figure 3:**
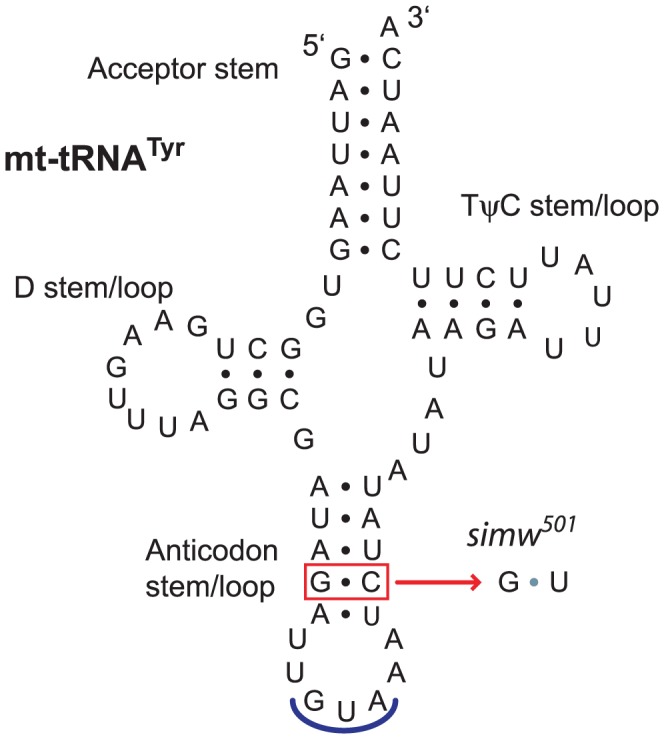
A mtDNA polymorphism in the *D. simulans* mt-tRNA^Tyr^ anticodon stem. The *D. simulans simw^501^* tRNA^Tyr^ has a G∶C to G∶U mutation in the anticodon stem relative to the *D. simulans sm21* and *D. melanogaster* mtDNAs. Shown is the *D. simulans sm21* sequence.

To identify the molecular basis for this mitochondrial-nuclear incompatibility, we first sequenced the incompatible *simw^501^* mtDNA and the closely related, compatible *D. simulans sm21* mtDNA, excluding the hypervariable control region. The two mtDNAs differ at only six positions out of 14,940 base pairs (Table 1): three synonymous SNPs located in *ATPase6*, *ND1*, and *ND5*, two changes at non-conserved sites in the lrRNA, and a SNP in the *tRNA^Tyr^* gene. The *tRNA^Tyr^* SNP is located at the base of the anticodon stem and changes a G∶C that is invariant within a population of *D. melanogaster* [36], [37] and conserved across the *D. melanogaster* species subgroup to a G∶U in *simw^501^* (Figure 3).

**Table 1:** Divergent sites between the *D. simulans sm21* and *simw^501^* mtDNAs.

		mtDNAs			
Locus	Position^1^	*sm21*	*simw^501^*	Other *D. mel* subgroup *spp.* 2	Consequence
*tRNA^Tyr^*	1433	G	A	Conserved G in subgroup	G∶C to G∶U in anticodon stem
*ATPase6*	4117	A	G	*D. mel, D. sec, D. mau*: A	Synonymous change
*ND5*	8136	A	G	*D. mel, D. sec, D. mau*: A	Synonymous change
*ND1*	12354	A	G	*D. mel, D. sec, D. mau*: A	Synonymous change
*lrRNA*	13163	A_10_	A_9_	*D. mel, D. sec, D. mau*: A_9_	Poly-A length change
*lrRNA*	14047	AGA	TAG	*D. mel*: TTT, *D. sec*: AAA, *D. mau*: TAA	Change at nonconserved position

1 Position using the *D. simulans* mitochondrial genome sequence AF200839.1 as a reference.

2 The sequence state in mtDNAs of the *D. melanogaster* species subgroup [34]. *D. mel* is *D. melanogaster* (NC_001709), *D. sec* is *D. sechellia* (NC_005780), and *D. mau* is the *D. mauritiana maII* haplotype (AF200830).

**Figure 4:**
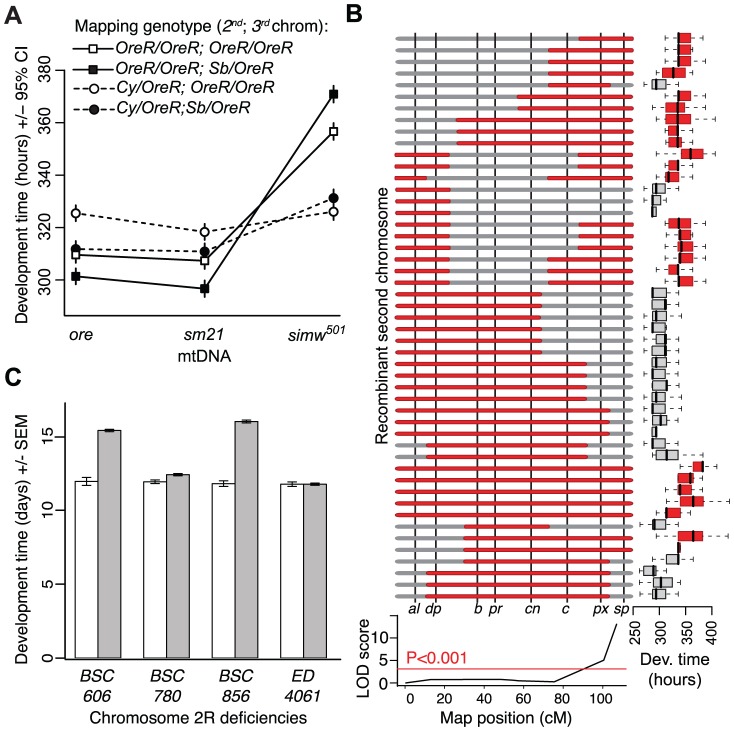
Genetic mapping implicates an interaction between the mt-tRNA^Tyr^ and its nuclear-encoded mt-TyrRS. (A) Chromosome segregation mapping using dominant, visible markers on the second and third chromosomes (*Cy* and *Sb*, respectively) revealed that the developmental delay is caused by a largely recessive factor on the *OreR* second chromosome. Only flies homozygous for the *OreR* second chromosome (squares) have extended development time. (B) Meiotic mapping using visible markers on the second chromosome indicates that flies with the *simw^501^* mtDNA that are homozygous for the *OreR* second chromosome (red) at the marker *speck* (*sp*) take significantly longer to develop than flies with the *simw^501^* mtDNA that are heterozygous for *OreR* and the mapping chromosome allele at this marker (gray), resulting in a significant marker-trait association (LOD score). (C) Two overlapping chromosomal deficiencies (*BSC606* and *BSC856*) at the tip of Chromosome arm 2R fail to complement the *OreR* nuclear factor and significantly extend development time when combined with the *simw^501^* mtDNA (*P_ANOVA_*<0.0001, both deficiencies). Two neighboring deficiencies (*BSC780* and *ED4061*) complement the nuclear factor and restore development time to that of controls. Gray bars represent mean development time of (*simw^501^*);*OreR* individuals inheriting the deficiency chromosome and white bars are control siblings inheriting a compatible balancer chromosome. The effects of these deficiencies are independent of sex, require the *simw^501^* mtDNA (Figure S2), and implicate *Aatm*, the only gene contained in both *BSC606* and *BSC856* with annotated mitochondrial function.

We used genetic mapping to identify the nuclear factor that interacts with the *simw^501^* mtDNA. Crosses between mitochondrial-nuclear genotypes revealed that the incompatible factor in the *D. melanogaster OreR* nuclear genome that delays development is autosomal and has largely recessive effects on development time (Figure 1B and 1C). Additional crosses using dominantly marked autosomes localized this factor to the second chromosome (Figure 4A). The effects of this mitochondrial-nuclear interaction on fecundity and bristle length also map to the second chromosome (Figure S1), but the pattern of dominance differs for these three phenotypes. The second chromosome genotype has a dominant effect on the reduction in fecundity in an *OreR* homozygous third chromosome background (Figure S1A) and a nearly additive effect on bristle length (Figure S1B). However, in all cases, the *D. melanogaster OreR* second chromosome has a negative phenotypic effect only when combined with the *simw^501^* mtDNA (i.e. the effects are always conditional on the mtDNA genotype).

### The Incompatible Nuclear Factor Is an Amino Acid Substitution in the Nuclear-Encoded *mt*-*Tyrosyl-tRNA Synthetase* Gene, *Aatm*

We used meiotic mapping with recessively marked second chromosomes to further localize the second chromosome factor affecting development time. Flies with the *simw^501^* mtDNA and homozygous for the *OreR* allele at the marker *speck (sp)* had significantly longer development time than individuals that were *OreR/sp* (*P_ANOVA_*<0.001), indicating that the factor is closely linked to *sp* at the tip of chromosome arm 2R (Figure 4B). Using chromosomes that contain overlapping deficiencies that span this segment of the genome, we localized the factor to a region containing nine genes that entirely account for the delayed development time when combined with the *simw^501^* mtDNA (Figure 4C and Figure S2). The only gene in this region with annotated mitochondrial function is the uncharacterized gene *CG16912*. The protein product of this gene is 47% identical and 65% similar to the human nuclear-encoded mitochondrial tyrosyl-tRNA synthetase (mt-TyrRS) encoded by *YARS2* [38], [39], and is predicted to have a mitochondrial-targeting signal by MITOPROT [40]. mt-TyrRS catalyzes the attachment of tyrosine to the mitochondrial-encoded tRNA^Tyr^ – the molecule containing one of the SNPs that differs between the *simw^501^* and *sm21* mtDNAs. We refer to the *D. melanogaster* gene encoding the aminoacyl-tRNA synthetase for tyrosine in the mitochondria as *Aats-tyr-m* (*Aatm*).

**Figure 5:**
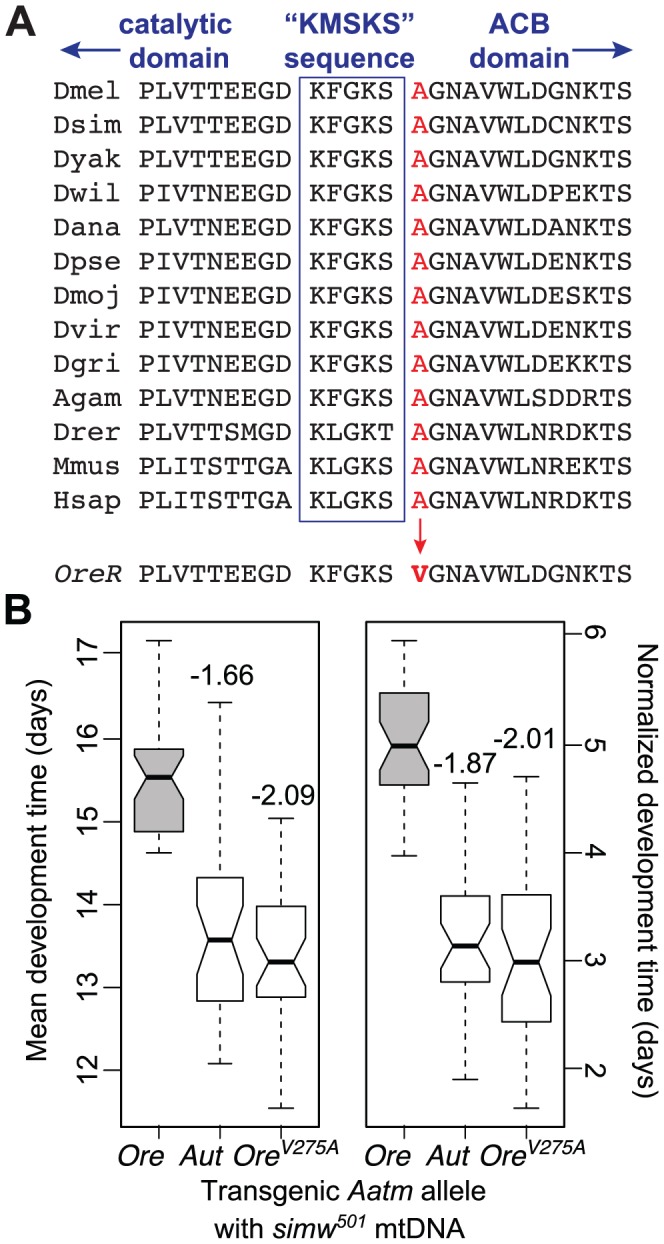
An amino acid change in the mt-TyrRS gene *Aatm* is incompatible with the *mt-tRNA^Tyr^* polymorphism. (A) A nonsynonymous SNP in the *D. melanogaster OreR* nuclear-encoded mt-TyrRS gene *Aatm* changes a highly conserved alanine to a valine at a residue adjacent to the class I aaRS “KMSKS” signature sequence located in a loop that connects the catalytic domain to the anticodon binding (ACB) domain [38]. Nine Drosophilid species sequences are followed by mosquito (*Anopheles gambiae*), zebrafish (*Danio rerio*), mouse (*Mus musculus*) and human. (B) Both the transgenic *Aatm^Aut^* allele and the *Aatm^Ore_V275A^* allele that reverts the valine in the *OreR* allele to the conserved alanine significantly recover development time in a *simw^501^* mitochondrial background and recapitulate the developmental difference between the (*simw^501^*);*OreR* and (*simw^501^*);*AutW132* genotypes. Boxplots show the distributions of mean development time for all individuals emerging from a single vial, and notches indicate the approximate 95% confidence intervals around the medians. Numbers above the boxes indicate the magnitude of the reduction in development time in days, relative to the incompatible *Aatm^Ore^* allele (*P_Tukey_*<0.0001, both alleles). Normalized development time is relative to control siblings that emerge from the same vial. Data were pooled across sexes, as the effects were the same in males and females (Figure S3 and Table S2).

We sequenced 2.3 kb containing the *Aatm* coding region and all intergenic nucleotides surrounding *Aatm* from the *OreR* and *AutW132* strains. The incompatible *OreR* allele (*Aatm^Ore^*) and the compatible *AutW132* allele (*Aatm^Aut^*) differ by one nonsynonymous SNP that changes a conserved alanine to a valine at amino acid position 275 (Figure 5A and Figure S3A) and one synonymous change that distinguishes *AutW132* from both the *OreR* and the *D. melanogaster* sequenced reference strain alleles. We used a transgenic approach to test whether the nonsynonymous A275V change in *Aatm^Ore^* causes the incompatibility with the *D. simulans simw^501^* mtDNA. We inserted three alleles of *Aatm – Aatm^Ore^*, *Aatm^Aut^*, and *Aatm^Ore_V275A^*, the *OreR* allele with the valine substitution changed to the conserved alanine (Figure S3) – into the same genomic location using ΦC31-mediated integration [41]. Due to the recessive nature of this interaction on development time, we tested the transgenic alleles in genotypes that carry the *simw^501^* mtDNA and a single copy of the endogenous *Aatm^Ore^* allele in *trans* to a deficiency that removes the homologous copy of *Aatm* (Figure S3B). Both the *Aatm^Aut^* and *Aatm^Ore_V275A^* alleles rescued the developmental delay in the *simw^501^* mitochondrial background (Figure 5B), and their effects were not significantly different from each other (Table S2). The difference in development time between flies expressing the incompatible *Aatm^Ore^* and the compatible *Aatm^Ore_V275A^* alleles in a *simw^501^* mitochondrial background is very similar to the two day difference in development time between (*simw^501^*);*OreR* and (*simw^501^*);*AutW132* flies (Figure 5B and Figure 1A). These results indicate that the *Aatm* A275V SNP in conjunction with the *D. simulans simw^501^* mtDNA *tRNA^Tyr^* stem SNP is responsible for the mitochondrial-nuclear incompatibility.

### Patterns of Decreased OXPHOS Activity Reveal Disrupted Mitochondrial Protein Synthesis

**Figure 6:**
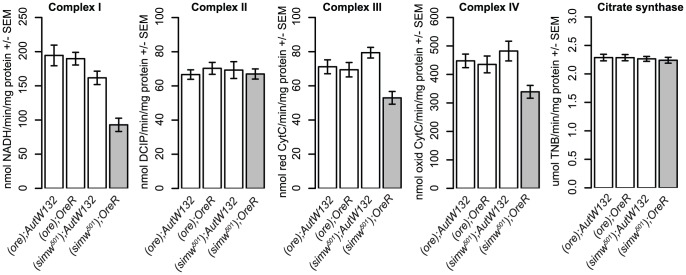
The mitochondrial-nuclear incompatibility decreases the activity of OXPHOS complexes that are mitochondrially translated. (*simw^501^*);*OreR* individuals have significantly reduced activities of OXPHOS complexes I, III and IV, which contain subunits encoded in the mtDNA and translated in mitochondria (mtDNA×nuclear, *P_ANOVA_*<0.01, each complex). In contrast, the activities of complex II and citrate synthase, which function in the mitochondria but are encoded entirely by nuclear genes and translated in the cytoplasm, are similar across genotypes (mtDNA×nuclear, *P_ANOVA_*>0.20, both activities). Plots combine male and female data, as the mitochondrial-nuclear interaction effects were the same in both sexes (Table S3).

The identity of these mutations suggested that mitochondrial translation is disrupted in (*simw^501^*);*OreR* individuals. tRNA mutations are known to disrupt stability, processing and aminoacylation of tRNAs [27], and similar mutations at the base of the anticodon stem of human and mouse mt-tRNA^Ile^ disrupt tRNA folding and decrease aminoacylation in cells [28]. Furthermore, the alanine at position 275 of mt-TyrRS is highly conserved and adjacent to the canonical “KMSKS” sequence located in the loop that connects the protein's catalytic domain to the tRNA anticodon-binding domain [38] (Figure 5A). A SNP in the human mt-TyrRS, *YARS2*, at an amino acid position that is conserved with *D. melanogaster* decreases aminoacylation efficiency via decreased rates of the reaction, as well as decreased affinity for tRNA^Tyr^ [39]. These data led us to hypothesize that the *simw^501^ mt-tRNA^Tyr^* and *Aatm^Ore^* SNPs interact to reduce the pool of tyrosine-charged tRNAs and compromise protein translation in the mitochondria. Consistent with this hypothesis, the activities of the OXPHOS enzyme complexes I, III, and IV, which contain subunits encoded by the mtDNA and translated in the mitochondria, are significantly decreased in (*simw^501^*);*OreR* adults (Figure 6 and Table S3). In contrast, the activities of complex II and citrate synthase, which function in the mitochondria but are encoded in the nuclear genome and translated in the cytoplasm, were not significantly affected (Figure 6 and Table S3), suggesting that mitochondrial abundance is similar among mitochondrial-nuclear genotypes.

## Discussion

We have shown that SNPs in the *D. melanogaster* nuclear-encoded mt-TyrRS gene *Aatm* and in the *D. simulans* mt-tRNA^Tyr^ have slight or no phenotypic effects in their native mitochondrial or nuclear backgrounds, but together these SNPs interact synergistically to severely decrease fitness via effects on larval development, metamorphosis, bristle development, and female fecundity. Below we discuss the physiology of larval development that we hypothesize underlies the observed fitness effects of compromised mitochondrial protein synthesis, and the implications of epistatic mitochondrial-nuclear interactions for the evolution of these genomes and the penetrance of mitochondrial disease.

### Cellular Physiology of the Mitochondrial–Nuclear Effect on Development

The mitochondrial-nuclear incompatible genotype that we have characterized specifically decreases the activity of OXPHOS complexes that contain subunits that are translated in the mitochondria, but not of Complex II and citrate synthase, both of which function in the mitochondria but are translated in the cytoplasm. This pattern indicates a disruption in either mitochondrial transcription or translation [25], [28], and suggests that mitochondrial abundance does not differ among our genotypes. Similar patterns of OXPHOS disruption in cells result from mutations at the base of the anticodon stem in human and mouse mt-tRNA^Ile^ that cause abnormal tRNA folding and inhibit aminoacylation [28]. Additionally, disease-associated mutations in the human mt-aaRS proteins encoded by *YARS2*, *DARS2* and *RARS2* all disrupt mt-tRNA aminoacylation [39], [42]. These observations, combined with the identity of the causal interacting mutations that we have identified, strongly indicate that decreased mitochondrial translation underlies compromised energetic function in the (*simw^501^*);*OreR* mitochondrial-nuclear genotype.

The bristle, development time, and female fecundity defects caused by this mitochondrial-nuclear incompatibility are strikingly similar to the classical *D. melanogaster Minute* mutants [43]. Those *Minute* mutants that have been mapped all disrupt components of the cytosolic ribosomes and are putatively unable to satisfy the protein synthesis demands of rapid growth during development [43], [44]. Other mutations that compromise cytoplasmic protein synthesis in *D. melanogaster*, such as those in the cytoplasmic aaRSs, also compromise bristle formation [45]. Our results demonstrate that disrupting the mitochondrial translational machinery phenocopies the *Minute* mutations, indicating that efficient mitochondrial function is required to energetically support cytoplasmic protein synthesis during larval growth and metamorphosis. Defective OXPHOS was an early hypothesis to explain the *Minute* phenotype [46], and disrupting cytochrome *c* oxidase activity in *Drosophila* yields small bristles [47], [48]. Furthermore, uncoupling of oxygen consumption from ATP production in *Drosophila* delays larval development and ovary maturation [43]. Thus, we hypothesize that the incompatibility between the *simw^501^* mtDNA and the A275V mutation in *Aatm^Ore^* results in an inability to energetically fund the “*Minute* reaction” [49].

The suite of phenotypes that result from both *Minute* mutations and the (*simw^501^*);*OreR* incompatibility suggests that specific cellular and developmental processes may be particularly sensitive to impaired protein synthesis. *Drosophila* larvae accumulate biomass in endoreplicating tissues that increase in size and ploidy during larval feeding in order to energetically support metamorphosis [50]. Starving larvae of amino acids arrests endoreplication in these tissues [51], suggesting that protein synthesis is a critical component of the endoreplicative increase in larval mass during development. Decreased mitochondrial activity may also slow larval development by inducing a starvation-like state in cells, as seen in the *D. melanogaster* bang sensitive mutants *kdn*, *tko* and *sesB* [52], [53], and via decreased cell proliferation, as seen in flies with mutations in the nuclear-encoded mt-metRS [30], the mitochondrial protein translocator component Tim50 [54], and subunit Va of cytochrome *c* oxidase [55]. These observations are consistent with the hypothesis that (*simw^501^*);*OreR* individuals are unable to energetically support adequate rates of protein sythesis for the genome endoreplication and cellular proliferation that is required for timely larval growth, metamorphosis and the construction of protein-rich adult structures such as bristles during development [43], [50], [56].

### Evolutionary Genetic Consequences of Epistasis between mtRNAs and tRNA Synthetases

Divergence in mtDNAs and at nuclear-encoded loci that function in the mitochondria may be driven by adaptive evolution of energy metabolism (e.g. [14], [57]) or by compensatory coevolution in response to slightly deleterious substitutions that accumulate in animal mtDNAs due to their high mutation rate, reduced effective population size, and lack of recombination [7], [9], [22]–[24]. Both of these processes are predicted to lead to the accumulation of coadapted sets of mitochondrial and nuclear substitutions in divergent lineages that may result in incompatibilities if these lineages hybridize [6]–[8], [10]. In the *D. melanogaster* species subgroup there is no evidence that coadapted mitochondrial and nuclear substitutions have accumulated as fixed differences between lineages [16]. This may result from a low mitochondrial substitution rate in *Drosophila* – approximately twice that of the nuclear genome – in contrast to other animal lineages where mitochondrial substitution rates can exceed nuclear rates by over 20-fold [9], [35], [58], [59]. The reason for the low rate of mtDNA substitution in *Drosophila* is unknown, but one possibility is a strong bias for G∶C to A∶T mutations in *Drosophila* mtDNA [60]. As more than 90% of third codon positions in *Drosophila* mtDNAs are A+T [35], the majority of mutations arising in a mutation accumulation experiment in *D. melanogaster* mtDNA occurred primarily at non-synonymous sites [60]. This mutation bias, combined with effective purifying selection due to the large effective population size of many *Drosophila* species, may account for the relatively low ratio of mtDNA∶nuclear substitution in *Drosophila* genomes. Evidence for coevolution of mitochondrial and nuclear genomes and mitochondrial-nuclear species incompatibilities comes primarily from lineages where substitution rates in the mitochondria are high, such as primates [9], [20], [21], *Tigriopus* [6] and *Nasonia* [61], [62], suggesting that the rate of mitochondrial substitution may be limiting for such compensatory coevolution to occur.

Nevertheless, the interaction that we have characterized demonstrates that *Drosophila* populations do harbor variation with the potential to generate mitochondrial-nuclear species incompatibilities. The incompatible A275V SNP in *Aatm^Ore^* is present at 13.2% in a North Carolina population of *D. melanogaster* [36], and the *simw^501^* mtDNA tRNA^Tyr^ G∶U segregates within the *D. simulans siII* mitochondrial haplogroup and is present in the *D. persimilis* sequenced strain [35]. These observations suggest that the polymorphisms that we have mapped are unlikely to be unconditionally deleterious. Variants such as these that have little effect on their own may drift to fixation in isolated lineages, and are precisely the type of substitutions that may lead to incompatibilities when isolated lineages hybridize. In insect lineages that accumulate deleterious mtDNA substitutions due to cytoplasmic sweeps driven by endosymbionts like *Wolbachia* [61]–[63] and in animal taxa that accumulate deleterious substitutions in mt-tRNAs due to high mutation rates and small effective population sizes [22], [23], we expect these mt-tRNA-nuclear tRNA synthetase interactions to coevolve via compensatory evolution and be a significant source of mitochondrial-nuclear hybrid incompatibilities.

### The Genetic Architecture of Mitochondrial Disease Phenotypes

The relationship between the mitochondrial genotype and the organismal phenotype is complex, due to the potential for heteroplasmy, interactions between mitochondrial and nuclear variants, and physiological homeostasis of energy metabolism in the cell. Homeostasis may mask the organismal phenotypic effects of genotypes that compromise mitochondrial function. For example, increased mitochondrial biogenesis compensates disrupted OXPHOS via a ROS-mediated signal in mouse cells with a mutation in the mt-tRNA^Ile^ [28]. Furthermore, different organismal phenotypes may be differentially sensitive to the effects of disrupted mitochondrial-nuclear function. The incompatibility described here manifests differently in three phenotypes, via different dosage effects of the incompatible *Aatm^Ore^* allele. When combined with the *simw^501^ mt-tRNA^Tyr^*, the effects of the *Aatm^Ore^* allele on female fecundity are largely dominant, while the effects on bristle development during metamorphosis and on larval development time are additive and recessive, respectively. This suggests that particular components of fitness may be differentially buffered against defects in mitochondrial protein synthesis, potentially as a result of different energy requirements for adult reproductive output versus development or a difference in the ability of the organism to modulate cellular homeostasis across different life stages or cell types.

Mitochondrial protein synthesis is a critical component of cellular and organismal health [64]. At least twelve human diseases have been associated with mutations in nuclear-encoded mt-aaRSs [30], and mutations in the human mt-TyrRS gene *YARS2* cause myopathy, lactic acidosis, and sideroblastic anemia [39]. Additionally, a large proportion of mitochondrial disease mutations are in mt-tRNAs [28], [29]. However, identical homoplasmic tRNA mutations can cause a diversity of clinical phenotypes with variable penetrance [27], [28], [64]–[67]. Cellular homeostasis has been proposed as one cause of variable penetrance [27], [28], [31]. This homeostasis may be achieved via increased mitochondrial biogenesis [28], compensatory expression of metabolic pathways [53], or increased expression of tRNA-interacting loci in the nuclear genome [67], [68]. In fact, increased expression of nuclear-encoded aa-RSs is known to modify disease state [67], [68]. A complementary explanation for variable disease penetrance is that segregating genetic variation at nuclear loci, such as those encoding the mt-aaRSs, modify the phenotypic effects of mutations in mitochondrial tRNAs. Evidence for this hypothesis has been lacking [27], [31], [66], with the exception of nuclear loci that modify the phenotypic effects of deafness-associated mitochondrial mutations in humans and mice [32], [33]. Our findings provide evidence that phenotypic effects of mutations in mitochondrial tRNA genes can be highly conditional on the nuclear background in which they are expressed, and may be particularly dependent on polymorphisms in their cognate nuclear-encoded aaRSs. Thus, the combined mitochondrial-nuclear genotype at tRNAs and their aaRSs may be a better predictor of disease than either genotype in isolation.

## Materials and Methods

### *Drosophila* Stocks and Maintenance

Mitochondrial genomes were introgressed by mating *D. simulans* females to hybrid rescue strains of *D. melanogaster* that produce fertile female offspring, which were then backcrossed to *D. melanogaster*. All nuclear genomes were precisely replaced with either *OreR* or *AutW132* chromosomes using non-recombining balancer chromosomes as described in [16]. Other cytoplasmic factors, such as *Wolbachia* and maternal piRNAs, were controlled for as described in [16]; as a result, no genotype carries *Wolbachia*. All flies were reared on standard media at 25°C with a 12 h∶12 h light∶dark cycle. Flies were collected using CO_2_ anesthesia and allowed to recover for a minimum of 24 hours before being used in experiments.

### Statistical Analyses

We used analysis of variance models described below for each trait to test for the effects of mtDNA, nuclear genotype and the mitochondrial-nuclear interaction, and we report *P-*values from these models using Type III sums of squares. We used Tukey's post-hoc contrasts to compare the phenotypic effects of transgenic alleles. All statistical analyses were done in the statistical package R version 2.13.0 [69]. Results of these analyses are presented in Tables S1, S2, S3.

### Development Time

For the initial chromosome segregation mapping experiment, we allowed females to lay eggs on grape-agar plates for 24 hours, collected 0–1 day old larvae using 20% sucrose in PBS, and placed 100 larvae in each of five vials per genotype. For all subsequent experiments, we allowed replicates of 5–10 pairs of parents of each cross or genotype to lay eggs in vials for 24 hours. After 24 hours, the flies were placed in a new vial to lay a second, and in some cases a third, brood of eggs. We scored the number of offspring emerging from each vial once a day. In a subset of experiments we also scored the day and time of first puparium formation. We measured development time for multiple broods from three to ten replicate groups of parents per genotype, yielding *N* = 9–20 replicate vials per genotype per experiment.

From these data, we analyzed the mean development time of all individuals emerging from a vial, as well as the time until the first individual emerged in each vial. In the deficiency mapping and transgenic allele experiments, each vial produces both experimental and control genotypes. In these experiments, we also analyzed the normalized mean development time of experimental genotypes by subtracting the development time of control siblings emerging from the same vial to account for vial-to-vial variation in development time. We fit analysis of variance models that included fixed effects of genotype, sex and brood, as well as all interactions. Occasionally, we removed a single outlier vial in which the development of flies of all genotypes was delayed when analyzing non-normalized development time data. However, when using normalized development time, these vials are no longer outliers. None of the significant genotype effects depended upon the measure of development time, data normalization, or the exclusion of outliers.

### Female Fecundity

We counted the number of eggs laid by fifteen individual females of each mitochondrial-nuclear genotype over the course of ten days. Single virgin females of each genotype were collected from multiple vials of density controlled cultures. These females were paired with two *(ore);OreR* males and allowed to lay eggs on grape-agar plates supplemented with active yeast paste. Each day, for ten days, females were transferred to a fresh plate and given new males, if needed, and the eggs on each plate were counted. Only females that were scored for the entire ten days were used for the statistical analysis, resulting in *N* = 7–14 females per genotype per experiment. We analyzed the total number of eggs laid per female using an analysis of variance model that included fixed effects of mtDNA, nuclear genotype and the mitochondrial-by-nuclear interaction. Fecundity was measured in two independent experiments: the first compared the four mitochondrial-nuclear genotypes in Figure 2A, and the second compared females emerging from the chromosome segregation mapping crosses and included the *sm21* mtDNA (Figure 2B and Figure S1A).

### Bristle Length

Posterior scutellar macrochaetae were measured using a Nikon dissecting microscope at 50× power (10× ocular, 5× zoom objective) with a micrometer scale built into the ocular lens. Five- to ten-day old adult flies were frozen at −20 °C and measured by orienting individuals in a bed of cotton so that the entire length of the bristle was in the focal plane. Bristles were measured from the tip to the base, where the bristle emerges from the scutellum. Ten to 14 flies of each sex were measured per genotype. Initial measurements recorded the left and the right bristle lengths. Because these did not differ significantly, one bristle per fly was subsequently measured. Independent measurements were made on separate generations of flies separated by several months. A subset of flies were also measured from the chromosome segregation mapping experiment to verify that the second chromosome factor affects bristle size. We analyzed bristle length data with an analysis of variance model that included the fixed effects of mtDNA, nuclear genotype, sex and all interactions.

### Genetic Mapping of the Nuclear Factor

We used development time as the phenotype to map the mitochondrial-nuclear interaction. Crosses between lines that carry the same mtDNA but different nuclear genomes (*OreR* and *AutW132*) were used to determine that the nuclear variant causing the incompatibility is autosomal and largely recessive (Figure 1B and 1C). *D. melanogaster* have two main autosomes (the second and third chromosomes) and a small dot chromosome (the fourth chromosome) that has very few genes. To localize the incompatibility to the second or third chromosome, we crossed *OreR* females carrying one of three mtDNAs – *ore*, *sm21*, *simw^501^* – to males that were heterozygous for the *OreR* autosomes and dominantly marked second (*Cy,Roi*) and third chromosomes (*Sb*). All X chromosomes in this mapping experiment were derived from *OreR*. The results indicated that one or more recessive factors on the second chromosome of *D. melanogaster OreR* are responsible for the extended development time (Figure 4A). We also measured fecundity of females of each genotype to verify that the second chromosome also affects this phenotype. Because *Sb* affects bristles, we only scored bristle lengths in flies homozygous for the *OreR* third chromosome. However, measuring bristle length in the remaining genotypes allowed us to confirm that the second chromosome factor also affects bristle length. Due to the strong effects of the second chromosome, we did not control the X or third chromosomes in the remainder of the mapping experiments. In these remaining mapping experiments, males inherit an *OreR* X chromosome, while females are heterozygous for an *OreR* X and an X that is either derived from or has potentially recombined with the mapping strain. Males and females are heterozygous for an *OreR* third chromosome and a third chromosome that is either derived from or has potentially recombined with the mapping strain.

We used meiotic mapping chromosomes carrying multiple recessive phenotypic markers (http://flystocks.bio.indiana.edu/Browse/misc-browse/mapping.htm) to map the factor on the second chromosome. We measured egg-to-adult development time of offspring inheriting a recombinant chromosome (between the *OreR* and the mapping chromosome) from their father and a *simw^501^* mtDNA and an *OreR* second chromosome from their mother. We localized the factor to the tip of chromosome arm 2R using a standard mapping analysis that calculates the likelihood of association (LOD score) between marker genotype and mean development time. Significance of the LOD score was determined by permutation using R/qtl version 1.23 [70].

We used chromosomes with molecularly defined deficiencies from the Bloomington *Drosophila* Stock Center [71] (http://flystocks.bio.indiana.edu/Browse/df/dfextract.php?num=all&symbol=bloomdef) to further localize the nuclear factor within the region mapped above. For each deficiency we crossed females of four genotypes – *(simw^501^);OreR*, *(simw^501^);AutW132*, *(sm21);OreR*, and *(ore);OreR* – to males carrying the deficiency chromosome over a balancer chromosome that carries a wild type and presumably compatible allele of the nuclear factor. We measured egg-to-adult development time of offspring inheriting either the deficiency or the balancer chromosome; flies carrying a deficiency that spans the target locus will be hemizygous for the *OreR* allele and are expected to have significantly extended development time in the *simw^501^* mitochondrial background. We iteratively used smaller and tiled deficiencies to refine the region containing the interacting nuclear locus. The smallest region of overlap between deficiencies that extended development time when paired with the *simw^501^* mtDNA contains only nine genes, one of which is *Aatm* (Figure S2).

### Sequencing

To identify differences between the *simw^501^* and *sm21* mtDNAs, we used standard PCR conditions and Sanger sequencing protocols. mtDNA primer sequences are provided in Table S4. The assembled mitochondrial sequences did not include the hypervariable control region. mtDNA sequences are deposited in GenBank as accession numbers KC244283 and KC244284. We used the primers listed in Table S5 to PCR amplify and sequence the *OreR* and *AutW132* alleles of *Aatm*, which differed by only two SNPs across a 2.3 kb region containing the coding region and all intergenic nucleotides (Figure S3A). The translations of these sequences were aligned using ClustalX to the amino acid sequences of nine Drosophilid species [72], as well as to the mosquito, zebrafish, mouse and human orthologs from GenBank. Using available sequences from the *D. melanogaster* genetic reference panel collected from a single population in Raleigh, North Carolina, USA [36], we found that 21/159 lines with an unambiguous SNP call at this position had the *Aatm^Ore^* A275V allele. We used available sequence data from the *Drosophila* Population Genomics Project [37] to determine that the *mt-tRNA^Tyr^* G∶C state was invariant within this same *D. melanogaster* population.

### Transgenic Rescue of the Mitochondrial–Nuclear Interaction for Development Time

A 2,326 bp genomic fragment containing the entire coding region of *CG16912* and extending into the flanking genes was PCR amplified from *OreR* and *AutW132* and cloned into a TOPO vector (Invitrogen, Carlsbad CA). The cloned fragments were sequenced, and mutations introduced by PCR were corrected with the QuickChange Lightning Site-Directed Mutagenesis kit (Agilent, La Jolla, CA). We used the same mutagenesis approach to construct a third allele that is identical to *OreR* except for a single T→C mutation that converts the valine at amino acid residue 275 to the conserved alanine (*Ore^V275A^*) (Figure S3A). These three alleles (*Ore*, *Aut*, and *Ore^V275A^*) were subcloned into the attB-P[acman] vector (generously provided by M. Cattani) and sequenced. We then inserted the transgenic constructs into the attP integration site VK00033 at cytological position 65B2 on the third chromosome of the *D. melanogaster* stock 9750 via ΦC31-mediated transgenesis [41]. Embryo injections were performed by BestGene (Chino Hills, CA). Transformed stocks were verified for insertion at 65B2 using a multiplex PCR reaction [73] with primers listed in Table S5.

We generated stocks that were homozygous for each transgenic allele on the third chromosome and heterozygous for a dominantly marked balancer second chromosome and a second chromosome carrying deficiency *Df(2R)BSC606* that spans *Aatm*. We crossed males from these three stocks to virgin *(simw^501^);OreR* females. Offspring from these crosses that inherit *Df(2R)BSC606* have a single transgenic allele of *Aatm* and a single endogenous *OreR* allele in the *simw^501^* mitochondrial background (Figure S3B). If a transgenic allele rescues the *(simw^501^);OreR* defect, then the development time of these flies should be ∼2 days shorter than that of flies carrying the *Aatm^Ore^* transgene. Control siblings inherit the compatible *Aatm* allele on the balancer chromosome and a single transgenic allele in the same mitochondrial background. To get normalized development time, we subtracted the mean development time of the control siblings from the experimental siblings that inherit the deficiency. Two independent replicate experiments using different generations of flies yielded the same result (Figure S3D). Comparisons among transgenic alleles were tested using Tukey's post-hoc contrasts.

### Mitochondrial Activity Assays

For measures of mitochondrial OXPHOS and citrate synthase activity, density-controlled male and female flies were aged separately for 15 days on standard media. Flies were gently homogenized in 1 mL chilled isolation buffer (225 mM mannitol, 75 mM sucrose, 10 mM MOPS, 1 mM EGTA, 0.5% fatty acid-free BSA, pH 7.2) using a glass-teflon dounce homogenizer. The extracts were centrifuged at 300 g for 5 minutes at 4°C. The supernatant was then centrifuged at 6,000 g for 10 minutes at 4°C to obtain a mitochondrial pellet. The pellet was resuspended in 100 µL of respiration buffer (225 mM mannitol, 75 mM sucrose, 10 mM KCl, 10 mM Tris-HCl, 5 mM KH_2_PO_4_, pH 7.2), aliquoted, and frozen at −80°C for enzyme activity assays. Protein was quantified in each mitochondrial sample using the BCA Protein Assay (Thermo Scientific, Rockford, IL, USA). We used these measures of protein abundance to standardize the amount of protein added to each reaction, and we optimized this amount separately for each enzyme assay. We assayed activity from six to eight biological replicates per sex for each genotype across either one or two blocks, with the activity of each biological replicate estimated from three technical replicate assays. We analyzed mitochondrial enzyme activities with analysis of variance models that included the fixed effects of mtDNA, nuclear genotype, sex, block and all interactions (Table S3).

The specific activity of complex I (NADH-ubiquinone reductase) was determined as the rotenone-sensitive rate, following the oxidation of NADH at 340 nm with the coenzyme Q analog decylubiquinone as the electron acceptor. The reaction mixture contained 35 mM NaH_2_PO_4_, 5 mM MgCl_2_, 2.5 mg/mL BSA, 2 mM KCN, 2 µg/mL antimycin A, 100 µM NADH, 100 µM decylubiquione and 15 µg mitochondrial protein, and was inhibited with 2 mM rotenone. The catalytic activity of complex II (succinate dehydrogenase) was monitored by the reduction of DCPIP at 600 nm. The reaction mixture contained 30 mM NaH_2_PO_4_, 100 µM EDTA, 2 mM KCN, 2 µg/mL antimycin A, 2 µg/mL rotenone, 750 µM BSA, 10 mM succinate, 100 µM DCPIP, 100 µM decylubiquinone and 15 µg mitochondrial protein, and was inhibited with 400 mM malonate. Complex III (cytochrome *c* reductase) activity was measured by monitoring the reduction of cytochrome *c* at 550 nm. The reaction mixture contained 35 mM NaH_2_PO_4_, 2.5 mg/mL BSA, 5 mM MgCl_2_, 2 mM KCN, 2 µg/mL rotenone, 50 µM cytochrome *c*, 25 µM decylubiquinol and 7 µg mitochondrial protein, and was inhibited with 5 µg/mL antimycin A. Potassium borohydride was used to reduce decylubiquione. Complex IV (cytochrome *c* oxidase) activity was measured by determining the rate of oxidation of reduced cytochrome *c* at 550 nm. The reaction mixture contained 5 mM MgCl_2_, 2 µg/mL Rotenone, 2 µg/mL Antimycin A, 1 mM DDM, 60 µM cytochrome *c* and 15 µg mitochondrial protein, and was inhibited with 4 mM KCN. Sodium dithionite was used to reduce cytochrome *c*. Equine heart cytochrome *c* was obtained from Sigma-Aldrich (C7752). To measure citrate synthase activity, the rate limiting reaction of citrate synthase was coupled to a chemical reaction in which DTNB reacts with CoA-SH and the absorbance of the product is measured at 412 nm. The reaction mixture contained 100 µM DTNB, 300 µM acetylCoA, 100 mM TrisHCl, 300 µM oxaloacetic acid and 6 µg mitochondrial protein.

## Supporting Information

Figure S1 The second chromosome interacts with the mtDNA to affect fecundity and bristle length. (A) The *OreR* second chromosome has a dominant effect to decrease fecundity, but this dominance effect is not observed in the *Sb* third chromosome background. This results in a significant three way interaction between the mtDNA and the autosomes. However, the presence of *Sb* has no effect when the *OreR* allele second chromosome is homozygous. There is little to no effect of autosomal genotype in the *ore* or *sm21* mitochondrial backgrounds. (B) The *OreR* second chromosome has an additive effect on bristle length in the *simw^501^* mitochondrial background (gray bars), but nuclear genotype has little to no effect in the *ore* or *sm21* mitochondrial background, resulting in a significant mitochondrial-nuclear interaction. Note the overall main effect that the *simw^501^* mtDNA has on bristle length. There was no sex-by-genotype interaction, and sexes are pooled in this plot.
(PDF)

Figure S2 Deficiency mapping localizes the nuclear factor to a region of nine genes on chromosome 2R. (A) Quantitative complementation mapping reveals two overlapping deficiencies (blue) that significantly extend development time, but only in the *simw^501^* mitochondrial background. The region of overlap between these deficiencies (gray) contains only 9 annotated genes, including the nuclear-encoded mt-TyrRS gene, *Aatm*. (B) The large effect of these deficiencies on development time occurs only in the *simw^501^* mtDNA background and is much stronger in the *OreR* than in the *AutW132* nuclear background, as expected given the strong mitochondrial-nuclear interaction effect. Values indicate the difference in development time in days between progeny inheriting a deficiency chromosome (Df, colored bars) and siblings that inherited a compatible *Aatm* allele on the CyO balancer second chromosome (CyO, black and gray bars). **P_ANOVA_<0.001, *** *P*<2e-10, **** *P*<2e-16.
(PDF)

Figure S3 Details and repeatability of the transgenic rescue experiment. (A) The *D. melanogaster OreR* and *AutW132 Aatm* alleles differ by two SNPs, with a single nonsynonymous change at nucleotide position 824 (gray) distinguishing the incompatible *OreR* allele from the compatible *AutW132* allele and from the *D. melanogaster* sequenced reference strain (“Ref”). (B) Transgenic genotypes expressing the *Aatm^Aut^*, *Aatm^Ore^*, and *Aatm ^Ore_V275A^* alleles that were used to test for allelic effects of *Aatm* in the *simw^501^* mtDNA background. (C) Both the *Aut* and *Ore^V275A^* alleles significantly reduce development time in both sexes relative to the *Ore* allele (*P_Tukey_*<0.0001, both alleles and sexes). Development time of individuals inheriting each transgene and an *Aatm* deficiency were normalized to control siblings that inherited the same transgene and a compatible *Aatm* allele. (D) An independent replicate experiment with a different generation of flies yields the same result (*P_Tukey_*<0.0001, both alleles and sexes). The two transgene alleles did not differ significantly from each other in either experiment. The estimated effects of the transgenic *Ore^V275A^* and *Aut* alleles on development time relative to the *Ore* allele are indicated above the boxes and are very similar to the average 2.17 day difference in development time between the pure *(simw^501^);OreR* and *(simw^501^);AutW132* individuals.
(PDF)

Table S1 Analysis of variance for mitochondrial and nuclear effects on organismal traits.
(PDF)

Table S2 Analysis of variance of the effects of transgenic *Aatm* alleles on development time.
(PDF)

Table S3 Analysis of variance of mitochondrial and nuclear effects on mitochondrial enzyme activities.
(PDF)

Table S4 Primers used to amplify and sequence the *sm21* and *simw^501^* mtDNAs.
(PDF)

Table S5 Additional primers used in this study.
(PDF)
