# Supplementary figures and images for: An Incompatibility between a Mitochondrial tRNA and Its Nuclear-Encoded tRNA Synthetase Compromises Development and Fitness in Drosophila
Source: PLoS Genet. 2013 Jan 31;9(1):e1003238. doi: 10.1371/journal.pgen.1003238 (PMC3561102; doi:10.1371/journal.pgen.1003238)

Figure S1

A

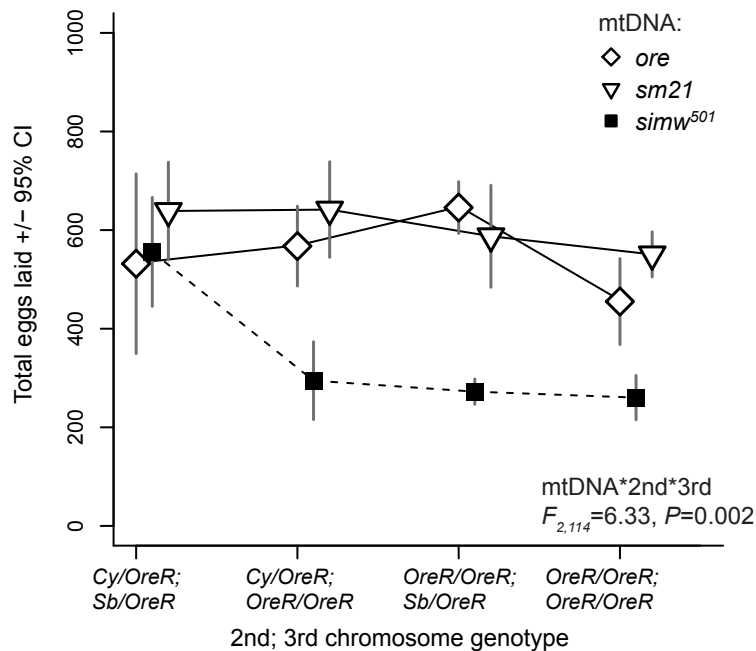

B

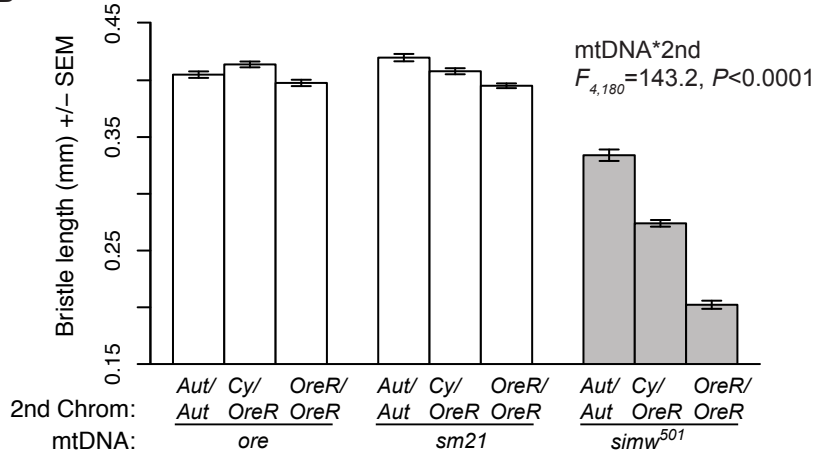

Supplement: Figure S1 — The second chromosome interacts with the mtDNA to affect fecundity and bristle length. (A) The OreR second chromosome has a dominant effect to decrease fecundity, but this dominance effect is not observed in the Sb third chromosome background. This results in a significant three way interaction between the mtDNA and the autosomes. However, the presence of Sb has no effect when the OreR allele second chromosome is homozygous. There is little to no effect of autosomal genotype in the ore or sm21 mitochondrial backgrounds. (B) The OreR second chromosome has an additive effect on bristle length in the simw501 mitochondrial background (gray bars), but nuclear genotype has little to no effect in the ore or sm21 mitochondrial background, resulting in a significant mitochondrial-nuclear interaction. Note the overall main effect that the simw501 mtDNA has on bristle length. There was no sex-by-genotype interaction, and sexes are pooled in this plot. (PDF) [file pgen.1003238.s001.pdf]

Figure S2

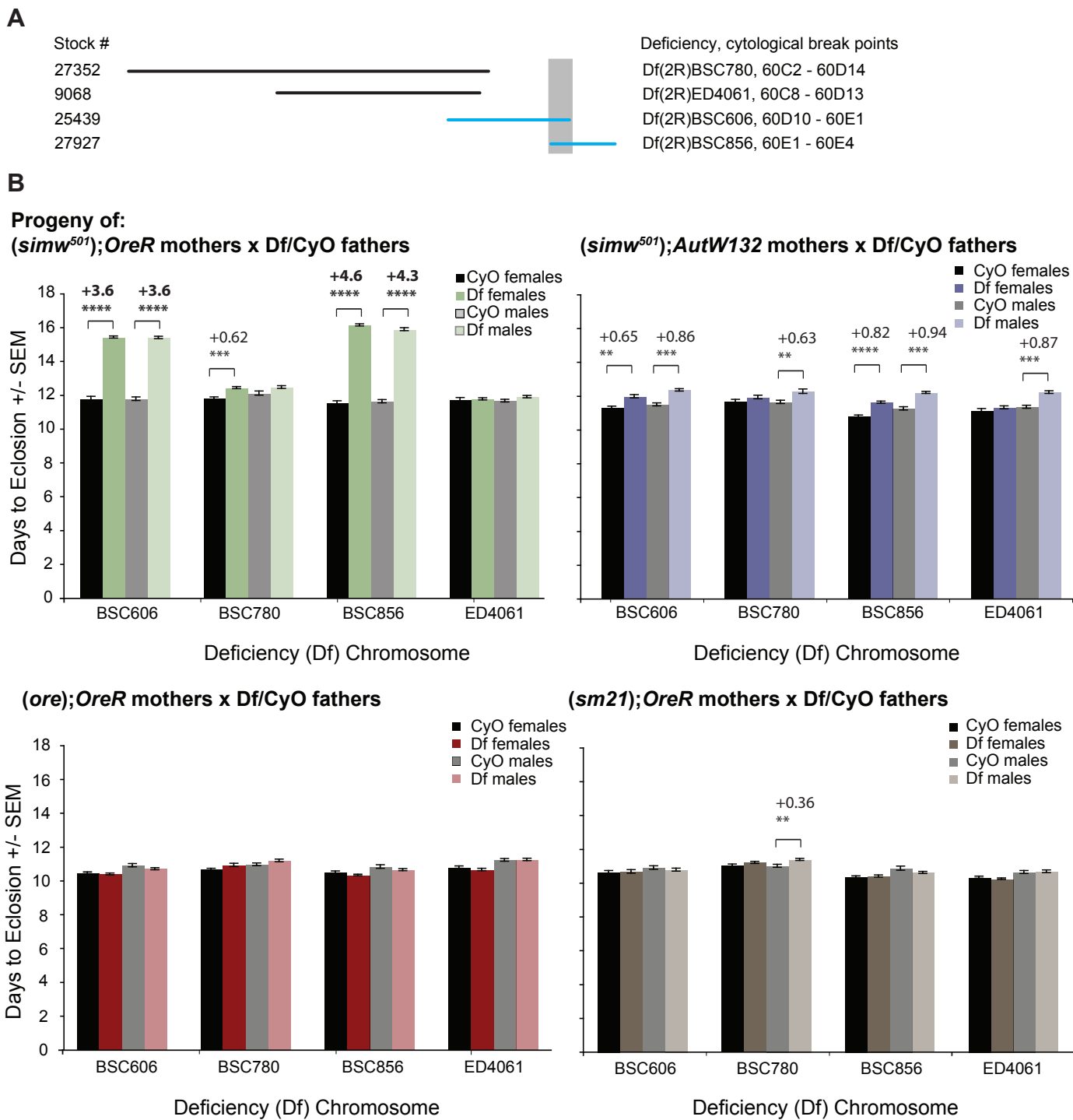

Supplement: Figure S2 — Deficiency mapping localizes the nuclear factor to a region of nine genes on chromosome 2R. (A) Quantitative complementation mapping reveals two overlapping deficiencies (blue) that significantly extend development time, but only in the simw501 mitochondrial background. The region of overlap between these deficiencies (gray) contains only 9 annotated genes, including the nuclear-encoded mt-TyrRS gene, Aatm. (B) The large effect of these deficiencies on development time occurs only in the simw501 mtDNA background and is much stronger in the OreR than in the AutW132 nuclear background, as expected given the strong mitochondrial-nuclear interaction effect. Values indicate the difference in development time in days between progeny inheriting a deficiency chromosome (Df, colored bars) and siblings that inherited a compatible Aatm allele on the CyO balancer second chromosome (CyO, black and gray bars). **PANOVA<0.001, *** P<2e-10, **** P<2e-16. (PDF) [file pgen.1003238.s002.pdf]

Figure S3

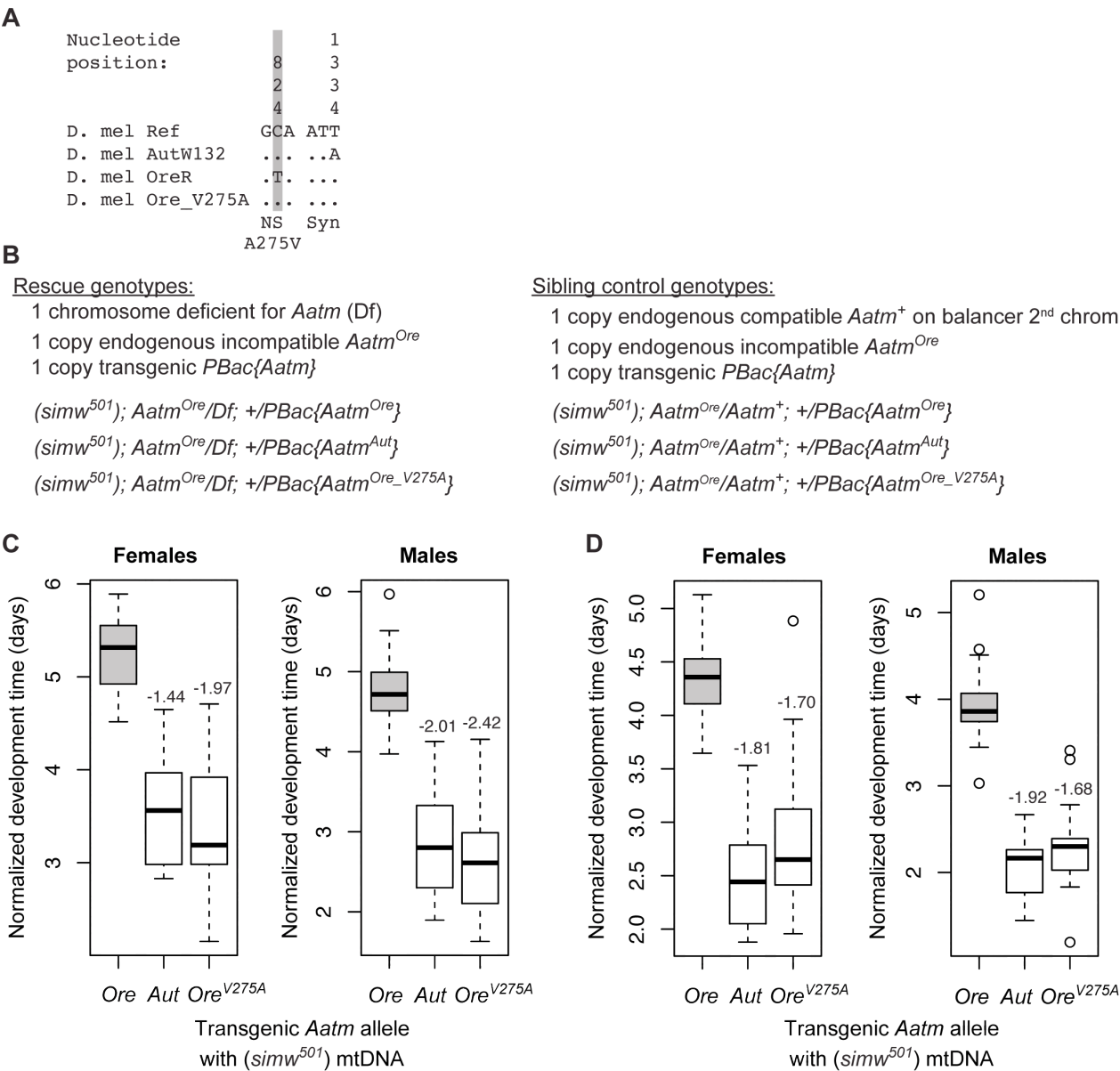

Supplement: Figure S3 — Details and repeatability of the transgenic rescue experiment. (A) The D. melanogaster OreR and AutW132 Aatm alleles differ by two SNPs, with a single nonsynonymous change at nucleotide position 824 (gray) distinguishing the incompatible OreR allele from the compatible AutW132 allele and from the D. melanogaster sequenced reference strain (“Ref”). (B) Transgenic genotypes expressing the AatmAut, AatmOre, and Aatm Ore_V275A alleles that were used to test for allelic effects of Aatm in the simw501 mtDNA background. (C) Both the Aut and OreV275A alleles significantly reduce development time in both sexes relative to the Ore allele (PTukey<0.0001, both alleles and sexes). Development time of individuals inheriting each transgene and an Aatm deficiency were normalized to control siblings that inherited the same transgene and a compatible Aatm allele. (D) An independent replicate experiment with a different generation of flies yields the same result (PTukey<0.0001, both alleles and sexes). The two transgene alleles did not differ significantly from each other in either experiment. The estimated effects of the transgenic OreV275A and Aut alleles on development time relative to the Ore allele are indicated above the boxes and are very similar to the average 2.17 day difference in development time between the pure (simw501);OreR and (simw501);AutW132 individuals. (PDF) [file pgen.1003238.s003.pdf]
