# Supplementary material for: An Incompatibility between a Mitochondrial tRNA and Its Nuclear-Encoded tRNA Synthetase Compromises Development and Fitness in Drosophila
Source: PLoS Genet. 2013 Jan 31;9(1):e1003238. doi: 10.1371/journal.pgen.1003238 (PMC3561102; doi:10.1371/journal.pgen.1003238)
Supplement: Table S1 — Analysis of variance for mitochondrial and nuclear effects on organismal traits. (PDF) [file pgen.1003238.s004.pdf]

Table S1. Analysis of variance for mitochondrial and nuclear effects on organismal traits

Dependent variable[1]	Factor	Df	*F* value	*P* value
Development time				
(mean emergence time)	**mtDNA**	**2**	**152.2**	**<2.2e-16**
N=10-20 replicate vials	**Nuclear**	**1**	**216.5**	**<2.2e-16**
	**Sex**	**1**	**6.631**	**0.0109**
	**mtDNA*nuclear**	**2**	**169.5**	**<2.2e-16**
	mtDNA*sex	2	0.041	0.9601
	**Nuclear*sex**	**1**	**6.309**	**0.0130**
	mtDNA*nuclear*sex	2	0.072	0.9307
	Residuals	164		
Development time				
(day of first emergence)	**mtDNA**	**2**	**185.7**	**<2.2e-16**
N=10-20 replicate vials	**Nuclear**	**1**	**465.2**	**<2.2e-16**
	**Sex**	**1**	**7.608**	**0.006**
	**mtDNA*nuclear**	**2**	**105.8**	**<2.2e-16**
	mtDNA*sex	2	0.761	0.469
	Nuclear*sex	1	0.579	0.448
	mtDNA*nuclear*sex	2	0.025	0.976
	Residuals	164		
Fecundity				
(total eggs over ten days)	**mtDNA**	**2**	**19.38**	**3.22e-07**
N=7-14 replicate females	**Nuclear**	**1**	**299.7**	**<2.2e-16**
	**mtDNA*nuclear**	**2**	**7.325**	**0.0014**
	Residuals	60		
Bristle length (mm)	**mtDNA**	**2**	**1372**	**<2.2e-16**
N=10-14 individuals	**Nuclear**	**1**	**501.3**	**<2.2e-16**
per sex per genotype	**Sex**	**1**	**37.03**	**1.30e-08**
	**mtDNA*nuclear**	**2**	**250.3**	**<2.2e-16**
	mtDNA*sex	2	0.470	0.6264
	Nuclear*sex	1	0.474	0.4925
	mtDNA*nuclear*sex	2	0.264	0.7680
	Residuals	126		

[1] Data include the following genotypes: *(ore);OreR*, *(ore);AutW132*, *(simw*^{501}*);OreR*, *(simw*^{501}*);AutW132*, *(sm21);OreR*, *(sm21);AutW132*.
