# Supplementary material for: An Incompatibility between a Mitochondrial tRNA and Its Nuclear-Encoded tRNA Synthetase Compromises Development and Fitness in Drosophila
Source: PLoS Genet. 2013 Jan 31;9(1):e1003238. doi: 10.1371/journal.pgen.1003238 (PMC3561102; doi:10.1371/journal.pgen.1003238)
Supplement: Table S2 — Analysis of variance of the effects of transgenic Aatm alleles on development time. (PDF) [file pgen.1003238.s005.pdf]

**Table S2.** Analysis of variance of the effects of transgenic *Aatm* alleles on development time

| Dependent Variable | Factor[1] | df | F | P | Contrasts | Effect | P |
|---|---|---|---|---|---|---|---|
| Mean development time | *Aatm* allele | 2 | 62.2 | < 2.2 e-16 | *Ore-Aut* | +1.66 days | <0.0001 |
| | | | | | *Ore-Ore*$^{V275A}$ | +2.09 days | <0.0001 |
| | | | | | *Aut-Ore*$^{V275A}$ | | NS |
| | Sex | 1 | 7.83 | 0.0063 | | | |
| | Brood | 2 | 5.30 | 0.0068 | | | |
| | Allele*brood | 4 | 3.64 | 0.0087 | | | |
| | Residuals | 86 | | | | | |
| Mean development time (normalized to control siblings) | *Aatm* allele | 2 | 180.9 | < 2.2 e-16 | *Ore-Aut* | +1.87 days | <0.0001 |
| | | | | | *Ore-Ore*$^{V275A}$ | +2.01 days | <0.0001 |
| | | | | | *Aut-Ore*$^{V275A}$ | | NS |
| | Sex | 1 | 40.0 | 9.66e-09 | | | |
| | Brood | 2 | 16.1 | 1.09e-06 | | | |
| | Allele*brood | 4 | 3.50 | 0.01052 | | | |
| | Residuals | 90 | | | | | |
| Day of first emergence | *Aatm* allele | 2 | 75.9 | < 2.2 e-16 | *Ore-Aut* | +1.73 days | <0.0001 |
| | | | | | *Ore-Ore*$^{V275A}$ | +1.94 days | <0.0001 |
| | | | | | *Aut-Ore*$^{V275A}$ | | NS |
| | Sex | 1 | 3.18 | 0.0782 | | | |
| | Allele*brood | 4 | 4.75 | 0.0017 | | | |
| | Residuals | 86 | | | | | |
| Day of first emergence (normalized to control siblings) | *Aatm* allele | 2 | 82.2 | < 2.2 e-16 | *Ore-Aut* | +1.67 days | 0.0001 |
| | | | | | *Ore-Ore*$^{V275A}$ | +1.83 days | 0.0001 |
| | | | | | *Aut-Ore*$^{V275A}$ | | NS |
| | Sex | 1 | 25.2 | 2.59e-06 | | | |
| | Allele*brood | 4 | 3.03 | 0.0217 | | | |
| | Residuals | 90 | | | | | |

[1] The full model contains the fixed effects of *Aatm* allele, sex and brood, and their interactions. Only significant factors are reported with Tukey's contrasts for allelic effects.
