# Supplementary material for: An Incompatibility between a Mitochondrial tRNA and Its Nuclear-Encoded tRNA Synthetase Compromises Development and Fitness in Drosophila
Source: PLoS Genet. 2013 Jan 31;9(1):e1003238. doi: 10.1371/journal.pgen.1003238 (PMC3561102; doi:10.1371/journal.pgen.1003238)
Supplement: Table S3 — Analysis of variance of mitochondrial and nuclear effects on mitochondrial enzyme activities. (PDF) [file pgen.1003238.s006.pdf]

Table S3. Analysis of variance of mitochondrial and nuclear effects on mitochondrial enzyme activities

Dependent variable[1]	Factor	Df	*F* value	*P* value
Complex I	**mtDNA**	**1**	**31.22**	**3.6e-06**
	**Nuclear**	**1**	**10.03**	**0.0034**
	Sex	1	1.971	0.1700
	Block	2	0.093	0.9118
	**mtDNA*nuclear**	**1**	**7.624**	**0.0095**
	mtDNA*sex	1	0.591	0.4480
	Nuclear*sex	1	1.796	0.1897
	mtDNA*block	2	0.318	0.7299
	Nuclear*block	1	1.247	0.3010
	mtDNA*nuclear*sex	1	0.700	0.4088
	mtDNA*nuclear*sex*block	1	0.444	0.6453
	Residuals	32		
Complex II	mtDNA	1	0.019	0.8913
	Nuclear	1	0.075	0.7859
	**Sex**	**1**	**13.33**	**0.0007**
	**Block**	**2**	**24.87**	**5.9e-08**
	mtDNA*nuclear	1	1.484	0.2296
	mtDNA*sex	1	1.165	0.2863
	**Nuclear*sex**	**1**	**8.322**	**0.0060**
	mtDNA*block	2	0.743	0.4815
	Nuclear*block	2	0.283	0.7546
	mtDNA*nuclear*sex	1	2.461	0.1239
	mtDNA*nuclear*sex*block	2	1.836	0.1715
	Residuals	44		
Complex III	mtDNA	1	1.282	0.2636
	**Nuclear**	**1**	**15.06**	**0.0003**
	Sex	1	0.513	0.4776
	**Block**	**1**	**5.791**	**0.0204**
	**mtDNA*nuclear**	**1**	**11.59**	**0.0014**
	mtDNA*sex	1	2.335	0.1337
	Nuclear*sex	1	0.102	0.7506
	mtDNA*block	1	2.527	0.1190
	Nuclear*block	1	0.575	0.4522
	mtDNA*nuclear*sex	1	0.116	0.7352
	mtDNA*nuclear*sex*block	1	1.236	0.2723
	Residuals	44		
Complex IV	mtDNA	1	2.291	0.1358
	**Nuclear**	**1**	**14.56**	**0.0003**
	**Sex**	**1**	**56.84**	**4.5e-10**
	**mtDNA*nuclear**	**1**	**10.25**	**0.0023**
	mtDNA*sex	1	0.329	0.5684
	Nuclear*sex	1	0.114	0.7364
	mtDNA*nuclear*sex	1	0.178	0.6747
	Residuals	56		
Citrate synthase	mtDNA	1	0.479	0.4954
	Nuclear	1	0.067	0.7979
	**Sex**	**1**	**4.640**	**0.0415**
	mtDNA*nuclear	1	0.050	0.8241
	mtDNA*sex	1	1.663	0.2095
	Nuclear*sex	1	0.528	0.4744
	mtDNA*nuclear*sex	1	0.131	0.7207
	Residuals	24		

[1] Data include the following genotypes: *(ore);OreR*, *(ore);AutW132*, *(simw*^{501}*);OreR*, *(simw*^{501}*);AutW132*.
