# Supplementary material for: An Incompatibility between a Mitochondrial tRNA and Its Nuclear-Encoded tRNA Synthetase Compromises Development and Fitness in Drosophila
Source: PLoS Genet. 2013 Jan 31;9(1):e1003238. doi: 10.1371/journal.pgen.1003238 (PMC3561102; doi:10.1371/journal.pgen.1003238)
Supplement: Table S4 — Primers used to amplify and sequence the sm21 and simw501 mtDNAs. (PDF) [file pgen.1003238.s007.pdf]

Table S4. Primers used to amplify and sequence the *sm21* and *simw*[501] mtDNAs

Forward primers[1]	Sequence	Reverse primers[1]	Sequence
47	ggattaccttgatagggtaaatcatgc	1112	gcagaataacagattcg
339	ggttaggagcttgaatagg	1461	cgcggttattgattaagtggc
1005	ggaggactacccccatttttagg	1930	gctcctagaattgaagaaattcc
TyrF_1325	agccttagtaaaaattactccttca	TyrR_1561	gttccgactatcccagctca
1852	cggagctggaacaggatgaactg	2827	gttgatccaatagtagacac
2783	ggattagctggaatacctcg	3647	ccataaaataaacctggtcg
3593	gaacagttcccgctttaggag	4528	gcagttaatcggacagctaatgtccc
ATP6F_4248	gaccatcaggtcataatggatctac	ATP6R_4603	aggtcctgtatttcctaaaagagtt
4463	gctcacttagtccctcaagg	5442	catgcagctgcttcaaatcc
5314	gctccatttactattgcggactc	6195	cattaacagtgatacgcctc
6143	gaagcgattgattgcagttagtttcg	6969	ctggggatttaaattgtgg
6931	accctaatattccacgaag	7740	atcgaattggagatgtagc
7852	gtcctaatccatctcatcc	8896	gggttattacgagttattgc
8780	gagcaacagatgaataagc	9672	gagtatgtgagggagctttagg
9699	gctccctcacatactctaaatg	10600	ggagctggtaaatctactaaagcg
10175	gggaggaatacttgtgttatttat	10712	cagcagtatagtgtatagc
10562	gctttagtagatttaccagctcc	11041	tcaacggcaaacccacctca
10641	ccagctccaattaatatctc	11573	ggttcttcgactggtcgagctcc
11355	gctaatcctttagttacacctgc	12329	gctggttgatcttctaattc
12312	gagctacagctcgtaaacctcc	13356	gccgcagtattttgactgtgc
12950	tccaacatcgaggtcgcaat	13294	ggctggaatgaatggttggacg
13321	cgtccaaccattcattccagc	14251	atgtacacatcgcccgtcgc
13888	aacgctgatacacaaggtacaa	14236	cacatcgcccgtcgctctta
14226	ccagtacatctactatgttacg	14787	ccaaattggtgccagcagtcgcgg
		srRNA_14929	ttaaagttttattttggc

[1] Primer names indicate approximate bp position in *D. simulans* mtDNA sequence AF200839.1
