# Supplementary material for: An Incompatibility between a Mitochondrial tRNA and Its Nuclear-Encoded tRNA Synthetase Compromises Development and Fitness in Drosophila
Source: PLoS Genet. 2013 Jan 31;9(1):e1003238. doi: 10.1371/journal.pgen.1003238 (PMC3561102; doi:10.1371/journal.pgen.1003238)
Supplement: Table S5 — Additional primers used in this study. (PDF) [file pgen.1003238.s008.pdf]

Table S5. Additional primers used in this study

Genomic region	Primer name	Primer sequence	PCR product (bp)	Primers used for sequencing
Aatm (Chrom 2R)	1909_F1<br>2202_R1<br>2411_R2	AAGAGCGGCAAGTTTTACGA<br>TAAACGCAGGCGGATTTTAC<br>TAACAAGACCTCGCCTTTCG	854	1909_F1, 2202_R1, 2722_R2
	2411_F2<br>3210_R3	GTCTGCTGGATCTCGGAAAG<br>ATCACGAGAACTGCCTTTGG	840	2411_F2, 3210_R3
	2923_F3<br>3708_R4	TTTGAAAGCAGCAGTTGTGG<br>TTCCAACGAACTTCGCATAA	826	2923_F3, 3708_R4
	3409_F4<br>4233_R5	CAGTGGATCAGGCCCATTAT<br>GATGTGTGGTTGCCTCGAC	863	3409_F4, 4233_R5
ΦC31-mediated insertion PCR	attP-F<br>attP-R	AGGTCAGAAGCGGTTTTCGGGAGTAGTG<br>GGTCGTAAGCACCCGCGTACGTGTCCAC	134	
	P[acman]-F<br>P[acman]-R	ACGCCTGGTTGCTACGCCTGAATAAGTG<br>CCCACGGACATGCTAAGGGTTAATCAAC	227 & 454	
